# Effects of Increased Summer Precipitation and Nitrogen Addition on Root Decomposition in a Temperate Desert

**DOI:** 10.1371/journal.pone.0142380

**Published:** 2015-11-06

**Authors:** Hongmei Zhao, Gang Huang, Yan Li, Jian Ma, Jiandong Sheng, Hongtao Jia, Congjuan Li

**Affiliations:** 1 Xinjiang Key Laboratory of Soil and Plant Ecological Processes, College of Grassland and Environmental Sciences, Xinjiang Agricultural University, Urumqi, China; 2 State Key Laboratory of Desert and Oasis Ecology, Xinjiang Institute of Ecology and Geography, Chinese Academy of Sciences, Urumqi, China; 3 Fukang Station of Desert Ecology, Chinese Academy of Sciences, Xinjiang, Fukang, China; 4 National Engineering Technology Research Center for Desert-Oasis Ecological Construction, Xinjiang Institute of Ecology and Geography, Chinese Academy of Sciences, Urumqi, China; Helmholtz Centre for Environmental Research (UFZ), GERMANY

## Abstract

**Background:**

Climate change scenarios that include precipitation shifts and nitrogen (N) deposition are impacting carbon (C) budgets in arid ecosystems. Roots constitute an important part of the C cycle, but it is still unclear which factors control root mass loss and nutrient release in arid lands.

**Methodology/Principal Findings:**

Litterbags were used to investigate the decomposition rate and nutrient dynamics in root litter with water and N-addition treatments in the Gurbantunggut Desert in China. Water and N addition had no significant effect on root mass loss and the N and phosphorus content of litter residue. The loss of root litter and nutrient releases were strongly controlled by the initial lignin content and the lignin:N ratio, as evidenced by the negative correlations between decomposition rate and litter lignin content and the lignin:N ratio. Fine roots of *Seriphidium santolinum* (with higher initial lignin content) had a slower decomposition rate in comparison to coarse roots.

**Conclusion/Significance:**

Results from this study indicate that small and temporary changes in rainfall and N deposition do not affect root decomposition patterns in the Gurbantunggut Desert. Root decomposition rates were significantly different between species, and also between fine and coarse roots, and were determined by carbon components, especially lignin content, suggesting that root litter quality may be the primary driver of belowground carbon turnover.

## Introduction

Climate models predict that arid regions of central Asia will experience enhanced intra- and inter-annual variability in the total amount of precipitation [1,2]. Nitrogen (N) application for agriculture has increased the rates of N deposition in arid ecosystems, and N deposition is predicted to increase in the future [3,4]. Given that water and N availability are the major limiting factors for biological activity, changes in both would likely influence litter decomposition in arid ecosystems [5,6].

Fine root production accounts for about 33% of the global annual net primary productivity, and is the most important component of total carbon (C) input [7,8]. Previous studies have focused on the influence of increased N deposition or altered precipitation regimes on fine root production and growth in arid ecosystems [7,9]. However, there are many uncertainties concerning decomposition of fine roots in arid lands. Consequently, it is crucial to elucidate the key controlling factors and processes of root decomposition in response to increasing rainfall and N deposition in order to understand the impact of changes on the C budget and nutrient cycling.

Although root systems store large amounts of plant biomass in desert ecosystems [10], few studies of litter decomposition have focused on roots compared with leaves [11–15]. Previous studies suggest that both climate factors and litter quality are important regulators of surface leaf litter decomposition at local and regional scales [14–17]. However, root decomposition rates do not mirror those of leaf litter in temperate ecosystems for several reasons [12,18,19]. First, the importance of tissue chemistry may differ between roots and leaf litter during decomposition, because root tissue commonly has lower C quality and a lower C:N ratio than is commonly observed in leaf litter [18]. Second, mycorrhizal fungi within and around root systems strongly alter root chemistry and architecture, and may thereby cause differences in decomposition of both litter types [20]. Third, belowground and aboveground environmental differences could overwhelm the effects of tissue chemistry on decomposition, because the belowground environment is more variable than the aboveground environment in terms of microclimate and soil biota [12,19].

To our knowledge, only a few experimental studies have been designed to identify predictors of root decomposition [15,18]. Limited research data have shown that tissue quality, N availability, rainfall pulses, and water penetration into the soil profile determine root litter mass loss in mesic and N-limited ecosystems, including temperate forests and grasslands [18,21–24]. Yet, some models of mesic ecosystems might be unsuited for analysis of deserts where plant productivity is limited by water availability and N; thus, ecosystem-specific characteristics may add complications to root decomposition [6,25]. In theory, litter should decompose at a slow rate in the desert due to its poor initial quality caused by low soil nutrient levels. However, studies have found that litter decomposes faster than expected in arid lands [26,27]. The rapid mass loss of aboveground litters may be due to abiotic factors, such as photodegradation, physical fragmentation, and leaching; however, few field experiments have addressed these factors for root decomposition [26,28].

In this study, we first monitored the decomposition process of some typical desert species with different initial chemical properties to elucidate the decomposition and nutrient release rate of roots in a typical temperate desert. Roots were categorized into fine (<2 mm) and coarse (>2 mm). Second, a manipulative experiment with N addition and increased summer precipitation was conducted to monitor the responses of desert root litter decomposition to projected environmental change. We hypothesized that: (1) simulative summer precipitation and N addition can accelerate the decomposition rate of roots in desert ecosystem due to the promotion of microbes; and (2) decomposition rates of fine roots should be higher than that of coarse roots, based on the lower C:N ratio of fine roots.

## Materials and Methods

### Ethics statement

The Fukang Desert Ecosystem Research Station is managed by the department of Xinjiang Institute of Ecology and Geography, Chinese Academy of Sciences. This study was approved by State Key Laboratory of Desert and Oasis Ecology, Xinjiang Institute of Ecology and Geography, Chinese Academy of Sciences and the Fukang Desert Ecosystem Research Station.

### Site description

This study was conducted in a long-term ecological research site of the Fukang Desert Ecosystem Research Station, Chinese Academy of Sciences (44°17′N, 88°56′E). The study area is located on the southern edge of the Gurbantunggut Desert. This region has a typical arid continental climate with dry hot summers and cold winters. According to climate data for the past 20 years, the annual mean temperature is 6.6°C, annual mean precipitation is 150 mm, and annual pan-evaporation is > 2000 mm [29]. Rainfall has strong annual and seasonal variability, with the majority falling in May–September. Soil is categorized into aeolian soil and alkaline soil, with coarse sand and high pH. The native vegetation is composed of desert shrubs (*Haloxylon ammodendron* and *Tamarix ramosissima*) and a herbaceous ground layer (*Reaumuria soongorica*, *Nitraria sibirica*, *Karelinia caspia*, *Salsola subcrassa* and *Suaeda acuminate*). In the experimental site, *H*. *ammodendron* dominates the plant community, and many ephemeral and ephemeral-like plants, such as *Erodium oxyrrhynchum*, *Lappula rupestris*, *Erysimum cheiranthoides*, *Eremurus inderiensis*, *Ephedra distachya* and the perennial herb *Seriphidium santolinum* proliferate in spring and early summer.

### Experimental design and treatments

#### Experiment Ι: Effects of water and N addition on decomposition dynamics of six desert herbaceous roots

Roots of six dominant herbs (*Phragmites communis*, *E*. *oxyrrhynchum*, *S*. *santolinum*, *S*. *subcrassa*, *K*. *caspia* and *N*. *sibirica*) were collected and used in our first decomposition experiment. The experiment used a randomized block design, with 12 plots and four treatments (control, water addition, N addition, and water plus N addition) distributed in three blocks. The area of each plot was 3 m × 3 m and plots were separated by a 1 m buffer belt.

We added N (granular urea in dry form) in mid-March 2011 and 2012, totaling 21 g N m^–2^ year^–1^. This amount of fertilizer was based on recommendations for alleviating N limitation in temperate deserts [4]. After N addition, the real amount of N enrichment in the site was approximately 7 g N m^–2^. For water addition treatments, 10 mm of tap water was manually applied with a sprayer in June, July, and August in 2011 and 2012. The total accumulation of water addition represented an increase of approximately 30% above mean annual rainfall in the decomposition plots. The amount of water addition was chosen to coincide with regional climate model predictions that climate change would increase summer precipitation more than 10% in northeastern and northwestern China [1,2].

The roots of *E*. *oxyrrhynchum*, *S*. *santolinum*, *S*. *subcrassa*, *P*. *communis*, *K*. *caspia* and *N*. *sibirica* were dug from the soil at their peak of growth in July–September 2010. The collected root materials were washed gently in tap water to remove adhering soil particles and organic debris, and only roots with diameter > 2 mm (coarse root) were selected for use in this experiment. The selected roots were air dried to a constant weight in the laboratory.

Roots samples were precisely weighed to 10.0 g and placed in 12 cm × 15 cm nylon litterbags composed of 0.1 mm mesh. Five subsamples of each litter type were oven-dried at 70°C for 48 h at the time of initial deployment to determine the initial dry mass and to analyze initial tissue chemistry. A total of 360 litterbags were prepared for decomposition. Roots litterbags were then horizontally buried at depths of 20 cm in the soil during October 2010. Litterbags were harvested after 0.5, 0.7, 1.0, 1.5, and 1.7 years of incubation. After retrieval, roots were removed from the bags, gently washed in tap water to remove adhering soil particles and organic debris, and oven-dried to a constant mass at 70°C to determine remaining dry mass. Corrections for inorganic contaminants were done after determining ash-free dry mass by incubating samples at 500°C for 4 h in a muffle furnace [30]. All mass and nutrient concentrations are reported on an ash-free basis.

**Fig 1:**
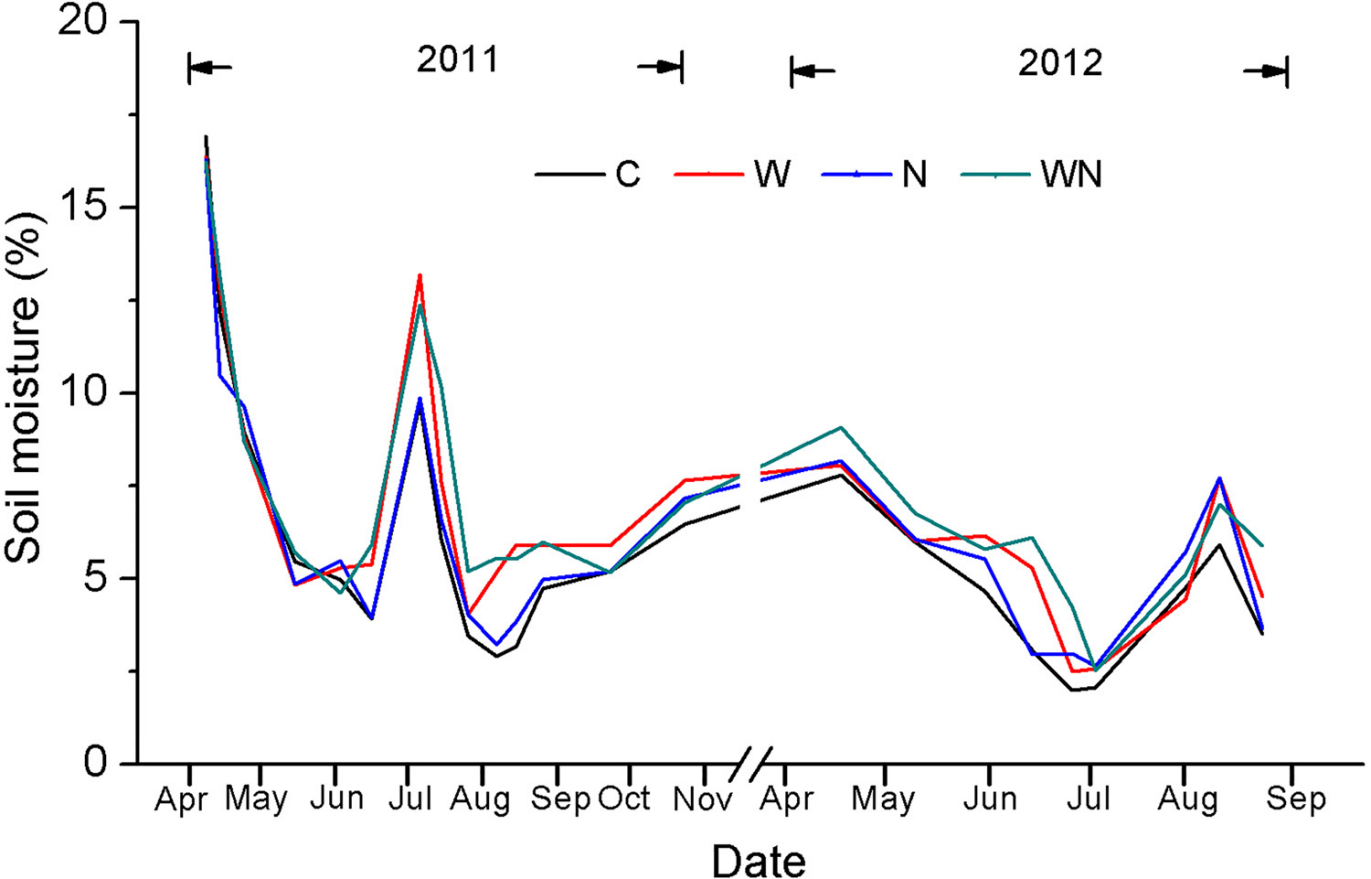
Seasonal changes in soil water content at the depth of 10 cm for control (C), water (W), nitrogen (N), and water plus nitrogen addition (WN) treatments (mean±SE).

**Fig 2:**
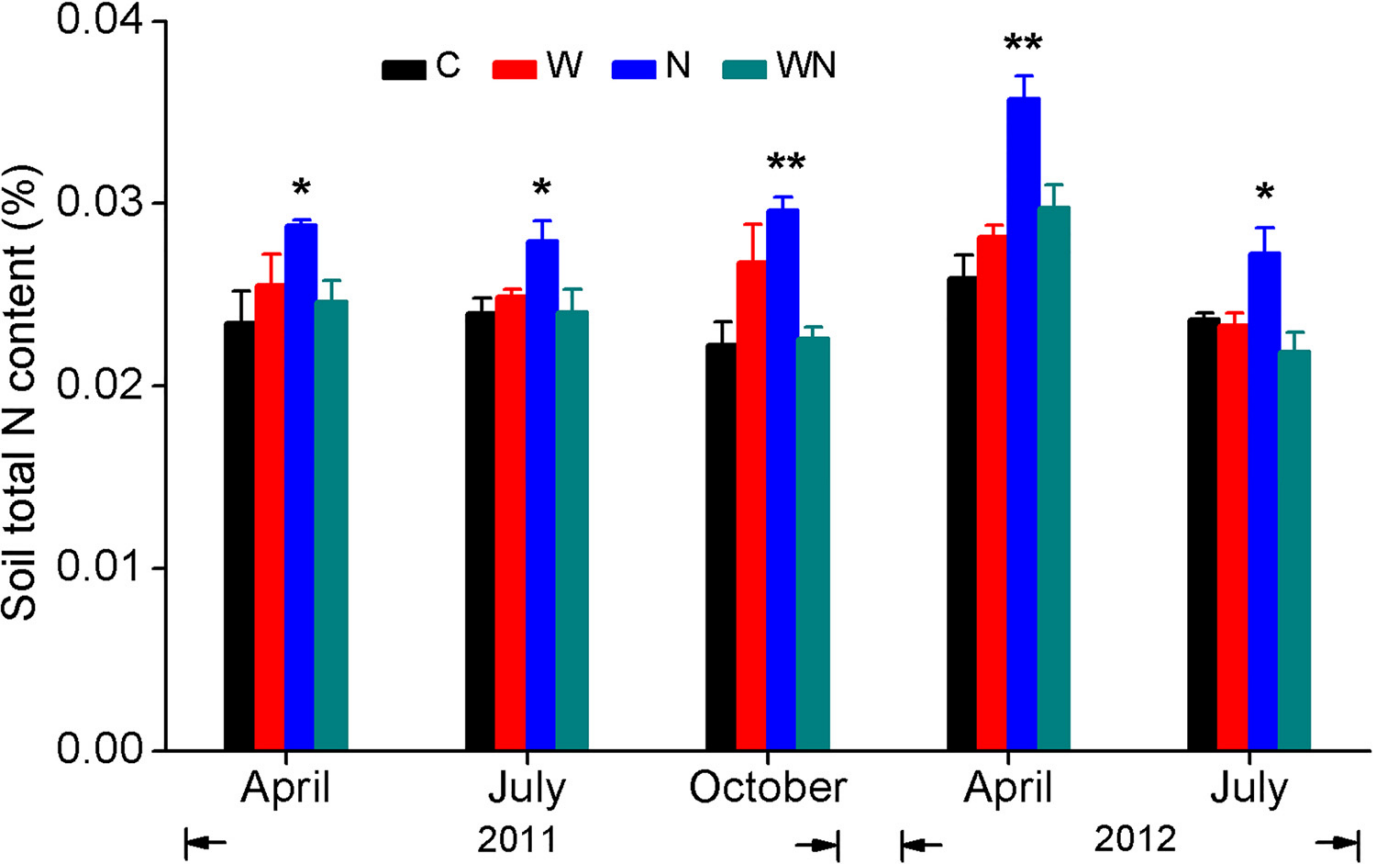
Soil total N concentration at the depth of 20 cm for control (C), water (W), nitrogen (N), and water plus nitrogen addition (WN) treatments (mean±*SE*). * and ** represent differences between the N addition treatment and control at *P*<0.05 and *P*<0.01, respectively.

Soil water content at 0–10 cm was measured using a weighing method at 10 d intervals from April to October in 2011 and 2012 (Fig 1). Soil samples at 20 cm depth were collected by hand auger in each plot at the same time as litter bag collection and then used to analyze soil total N content by the micro-Kjeldahl method (Fig 2).

#### Experiment II: Effect of root diameter on root decomposition

The second experiment examined whether root diameter class modified the effects of increased summer precipitation and N addition on root decomposition. Fresh roots of *E*. *oxyrrhynchum* and *S*. *santolinum* were categorized into fine (<2 mm) and coarse roots (>2 mm) according to root diameter, and then air-dried at room temperature to a constant mass. Root samples of 10.0 g of each diameter class were placed in litterbags (nylon, 12 cm × 15 cm). Sub-samples of each material were used to develop air-dried to oven-dried (70°C) conversions and were analyzed for initial dry mass and tissue chemistry. The litterbags were re-buried at depths of 20 cm in the original plots, which received identical patterns of water and N addition in October 2010. Three litterbags for each diameter class from each treatment plot were collected based on the sampling times of experiment Ι. On each harvest day, fine and coarse roots were removed from the bags, washed to remove extraneous matter, oven-dried to constant mass, weighed, ground, and milled for tissue chemistry analyses.

### Chemical analysis

All root samples were milled to powder for chemical analysis. Litter C content was measured using the K_2_Cr_2_O_7_ oxidation method [31]. Total N was determined by the semi-micro Kjeldahl method using an Alpkem semiautomatic analyzer (Kjektec System 1026 distilling unit, Sweden) [32]. Total phosphorus (P) was determined by colorimetric analysis (using a spectrophotometer UV–2401PC, Japan) with ammonium molybdate and ascorbic acid, after a sulfuric acid/hydrogen peroxide digestion [33]. Lignin was analyzed using the forage fiber technique [34]. Subsamples (0.5 g) were ground through a Wiley mill and subjected to sequential neutral detergent fiber, acid detergent fiber, and sulfuric acid (acid detergent lignin) digestions using an ANKOM semiautomatic fiber analyzer (ANKOM Technology, USA).

### Statistical analysis

Decomposition rate (*k*) was estimated using the negative exponential decay function of *X*_*t*_/*X*_*0*_ = *e* ^*−kt*^ [35], where *X*_*0*_ is the initial litter mass, *X*_*t*_ is the remaining litter mass after a given time period *t*, and *t* is decomposition time. *R*-squared values express the goodness of the model fit. The half-life (*t*_50%_) was calculated by 0.693/*k*. The remaining N or P at each harvest date was calculated by multiplying root mass remaining by its root N or P concentration and comparing it to the initial amount of litter root N or P [19].

For the first experiment, the test of significance of the initial litter chemistry, soil N content and decomposition rates, and residual N and P content were performed by analysis of variance (ANOVA). The Pearson-coefficient was used to examine the correlation between the initial chemical quality of the litter and the corresponding decomposition rates. Additionally, a model type II (standardized major axis, SMA) regression analysis was performed fitting log_e_ percentage mass remaining as a function of decomposition time using SMATR Version 2.0 [36]. Tests for slope heterogeneity among treatments followed the protocols of Warton and Weber [37]. In the second experiment, covariance analysis (ANCOVA) was used to test the effects of root diameter, as well as water and N addition on mass remaining, with measuring time as the covariate factor. A four-way ANOVA was used to test the effect of root diameter, species, water, and N addition on decomposition rate (*k*) and residual N and P content. Statistical analyses were conducted using SPSS 16.0 software (SPSS Institute Inc., Chicago, IL, USA). The least significant difference was used for comparisons of means with a confidence level of *P*<0.05.

## Results

### Soil moisture and nutrient conditions

Soil moisture in the water addition and water plus N addition treatments were 14.6 and 20.2% higher, respectively, than control treatments (C) (Fig 1), but the effect of the water addition on soil moisture was not significant (*F =* 2.758, *P =* 0.098). Also, N and water addition treatments had no significant effects on soil moisture (*F =* 0.058, *P =* 0.811). Addition of N significantly increased soil total N content by 24.6% (Fig 2).

### Experiment I: Effects of water and N addition on root decomposition

#### Effects of water and N addition on mass loss

Initial chemical content of litter indicates the litter quality of a species. All initial chemical parameters substantially differed among species except for litter C concentration (*P*<0.05, Table 1). Initial N content was highest in *N*. *sibirica* and lowest in *P*. *communis*. The variation of the C:N ratio was largely determined by changing patterns of N concentration. The initial lignin content had large variation among species, ranging from 33.9 mg g^–1^ in *P*.*communis* to 224.6 mg g^–1^ in *S*. *santolinum*. Similarly, the initial lignin:N of *S*. *santolinum* was nearly five times of that of *S*. *subcrassa*. The initial P content was similar among species, except for *P*. *communis* (Table 1).

**Table 1:** Initial litter chemistry of roots.

Species	C (mg g^–1^)	N (mg g^–1^)	P (mg g^–1^)	Lignin (mg g^–1^)	C:N	Lignin:N
***E*. *oxyrrhynchum***	**442.5** ^**a**^ **(22.1)**	**7.5** ^**a**^ **(0.2)**	**0.7** ^**a**^ **(0.0)**	**109.1** ^**a**^ **(10.1)**	**59** ^**a**^ **(5)**	**15** ^**a**^ **(2)**
***S*. *santolinum***	**468.4** ^**a**^ **(12.8)**	**7.8** ^**b**^ **(0.2)**	**0.7** ^**a**^ **(0.0)**	**224.6** ^**b**^ **(5.6)**	**60** ^**a**^ **(3)**	**29** ^**b**^ **(1)**
***S*. *subcrassa***	**417.3** ^**a**^ **(10.1)**	**6.6** ^**c**^ **(0.1)**	**0.8** ^**a**^ **(0.0)**	**39.4** ^**c**^ **(0.7)**	**63** ^**ac**^ **(3)**	**6** ^**c**^ **(0.2)**
***P*. *communis***	**457.4** ^**a**^ **(17.9)**	**4.2** ^**d**^ **(0.1)**	**1.4** ^**b**^ **(0.0)**	**33.9** ^**c**^ **(2.0)**	**110** ^**b**^ **(2)**	**8** ^**c**^ **(0.3)**
***K*. *caspia***	**475.1** ^**a**^ **(32.1)**	**6.4** ^**c**^ **(0.0)**	**0.8** ^**a**^ **(0.0)**	**96.0** ^**a**^ **(3.6)**	**74** ^**c**^ **(5)**	**15** ^**a**^ **(1)**
***N*. *sibirica***	**453.1** ^**a**^ **(46.8)**	**11.5** ^**e**^ **(0.4)**	**0.9** ^**a**^ **(0.0)**	**190.0** ^**d**^ **(0.9)**	**39** ^**d**^ **(3)**	**17** ^**a**^ **(1)**

Values given for C, N, P, lignin, C:N, and lignin:N ratios are means, with the standard errors given in parentheses.

Different superscript lowercase letters represent significant difference (*P*<0.05) among species.

**Fig 3:**
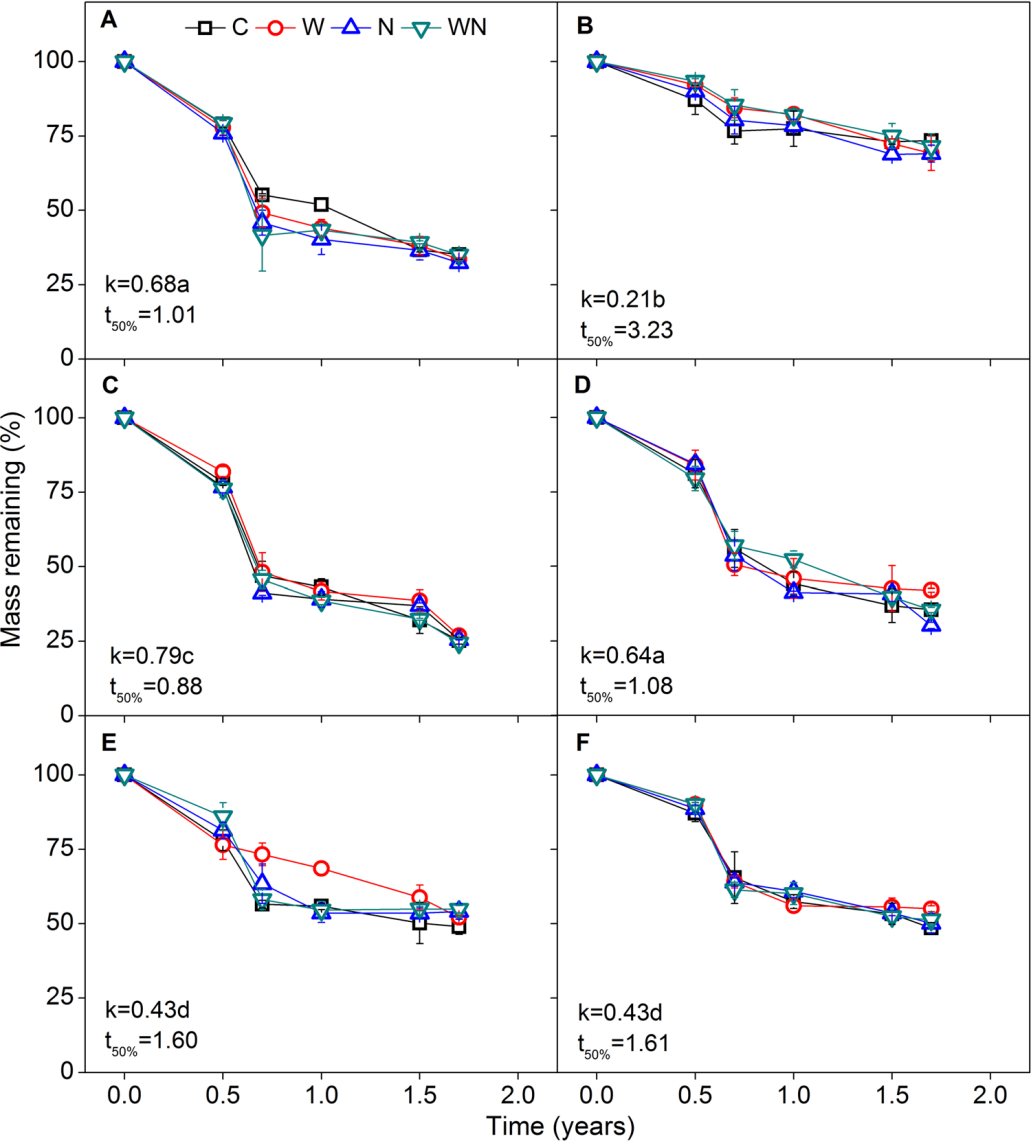
Residual mass of *E*. *oxyrrhynchum* (A), *S*. *santolinum* (B), *S*. *subcrassa* (C), *P*. *communis* (D), *K*. *caspia* (E), and *N*. *sibirica* (F) as affected by time after incubation for 1.7 years under control (C), water (W), nitrogen (N) addition, and water plus nitrogen additions (WN) in the field (experiment I). Values of *k* were determined using the negative exponential regression (*X*_*t*_/*X*_*0*_ = *e* ^*−kt*^) as proposed by Olson (1963). All *k* values present came from significant regression (*P*<0.01) with *R* ^*2*^ values within the range of 0.92–0.99. C, W, N, and WN treatments were averaged by species to calculate mean decomposition rates (*k*). Vertical bars represent standard errors (n *=* 3). Different lowercase letters indicate significant differences among species (*P*<0.05).

Litter mass loss of the six herb species showed similar patterns of change during the 1.7 years of decomposition, with high mass loss within the first 0.7 years followed by a stable phase (Fig 3). The mass loss curves were well fitted by one-exponent negative exponential models (*R* ^*2*^ *=* 0.92–0.99). Species showed different rates of mass loss after 1.7 years of decomposition, and mean mass loss of *S*. *subcrassa* (74.5%) was highest, followed by *E*. *oxyrrhynchum* (66.0%), *P*. *communis* (64.2%), *N*. *sibirica* (48.8%), *K*. *caspia* (47.5%), and *S*. *santolinum* (29.2%).

The SMA regression indicated no significant effect of water and N addition on decomposition curves of the six species (i.e. slope and *y*-intercept of the linear regression lines among treatments were equal, *P*>0.05) and the decomposition rate constants (*k*-values) were similar among treatments. However, significant differences in decomposition rates occurred among species within treatments (*P*<0.05), and litter with high quality decomposed faster than that of low quality, such that *S*. *santolinum* had the lowest *k*-value and *S*. *subcrassa* the highest (Fig 3).

#### Effects of water and N addition on nutrient release

**Fig 4:**
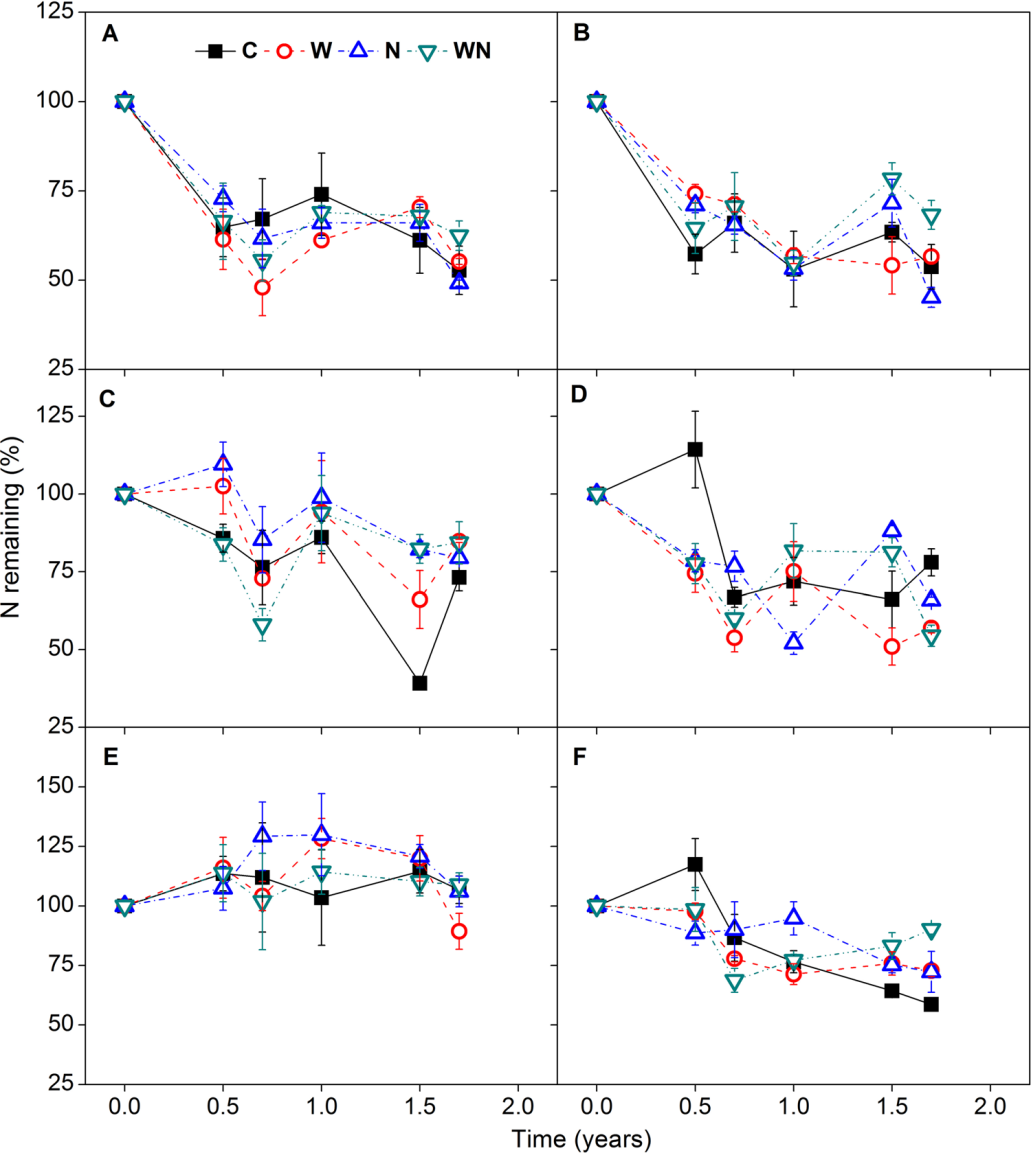
Residual N (as percentage of initial content) of *E*. *oxyrrhynchum* (A), *S*. *santolinum* (B), *S*. *subcrassa* (C), *P*. *communis* (D), *K*. *caspia* (E), and *N*. *sibirica* (F) during the decomposition process under control (C), water (W), nitrogen (N), and water plus nitrogen addition (WN) treatments (experiment I). Vertical bars represent standard errors (n = 3).

All species showed a net release of N after 1.7 years of decomposition except for *K*. *caspia* (Fig 4). Immobilization of N was found during decomposition of *S*. *subcrassa*, *P*. *communis*, *K*. *caspia*, and *N*. *sibirica*. Similar to mass loss, N loss was also initially rapid (as observed at 0.7 years), and during this initial period, water and N addition did not have any significant effects on N loss. The addition of N significantly affected N loss of *S*. *subcrassa*, *P*. *communis* and *N*. *sibirica* after 0.5 years of decomposition. The N remaining at 1.7 years was significantly lower in the control treatment than in the N-addition treatment for *S*. *subcrassa* (*F =* 7.45, *P<*0.05). However, water addition only significantly decreased the N remaining at 1.7 years for *P*. *communis* compared with control (*F =* 20.66, *P<*0.05, Fig 4).

**Fig 5:**
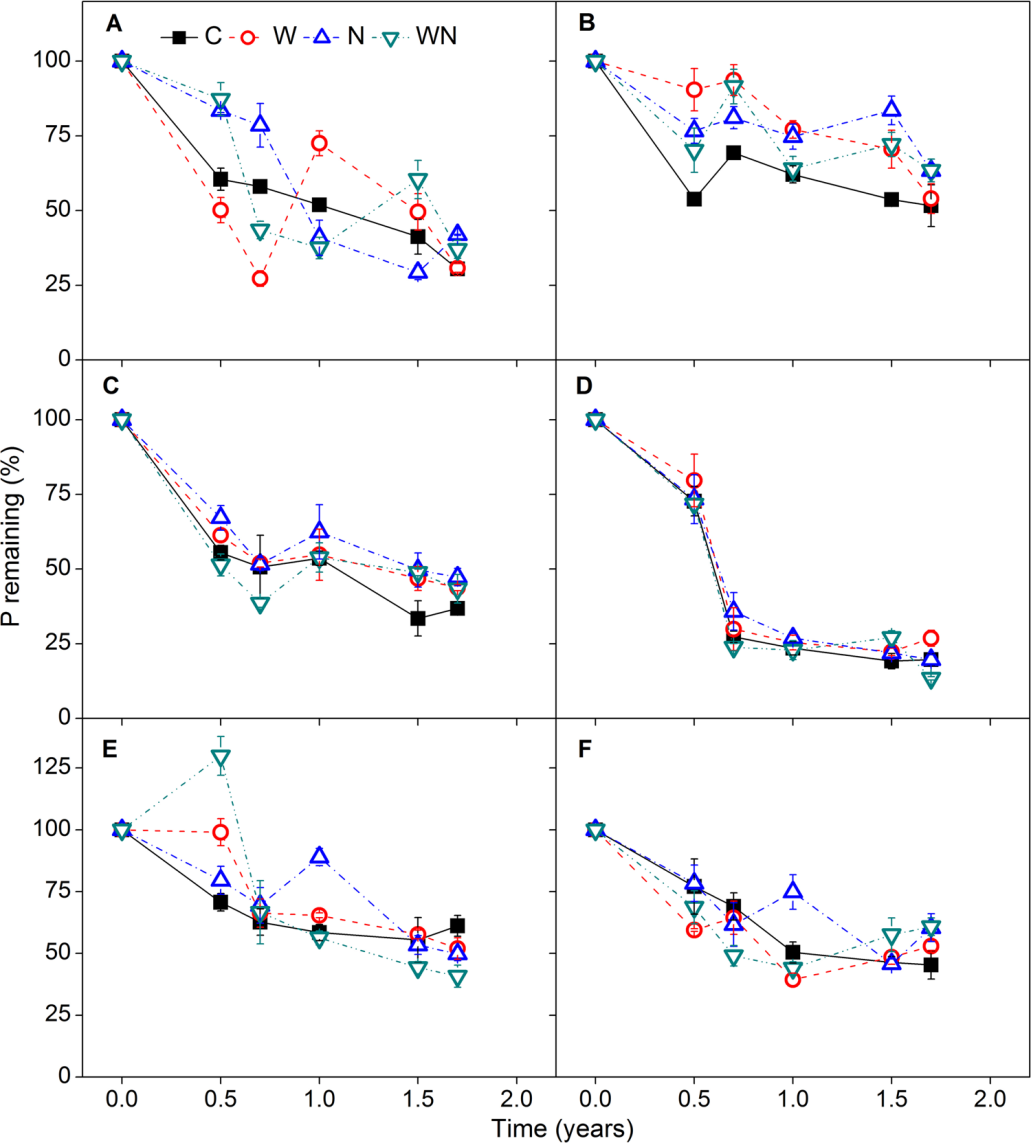
Residual P (as percentage of initial content) of *E*. *oxyrrhynchum* (A), *S*. *santolinum* (B), *S*. *subcrassa* (C), *P*. *communis* (D), *K*. *caspia* (E), and *N*. *sibirica* (F) during the decomposition process under control (C), water (W), nitrogen (N), and water plus nitrogen addition (WN) treatments (experiment I). Vertical bars represent standard errors (n = 3).

Similarly, all species showed a net release of P after 1.7 years of decomposition (Fig 5). Loss of P was initially rapid and N addition had no significant effect on P loss after 0.5 years, except for *E*. *oxyrrhynchum* and *S*. *santolinum*. Litter P remaining after 1.7 years of decomposition was lower in the control than for the water addition (*F =* 21.80, *P =* 0.01) and N addition (*F =* 11.05, *P =* 0.03) treatments for *S*. *subcrassa* (Fig 5).

### Experiment II: The critical role of root diameter on root decomposition

#### Variations of mass loss between fine and coarse roots

**Fig 6:**
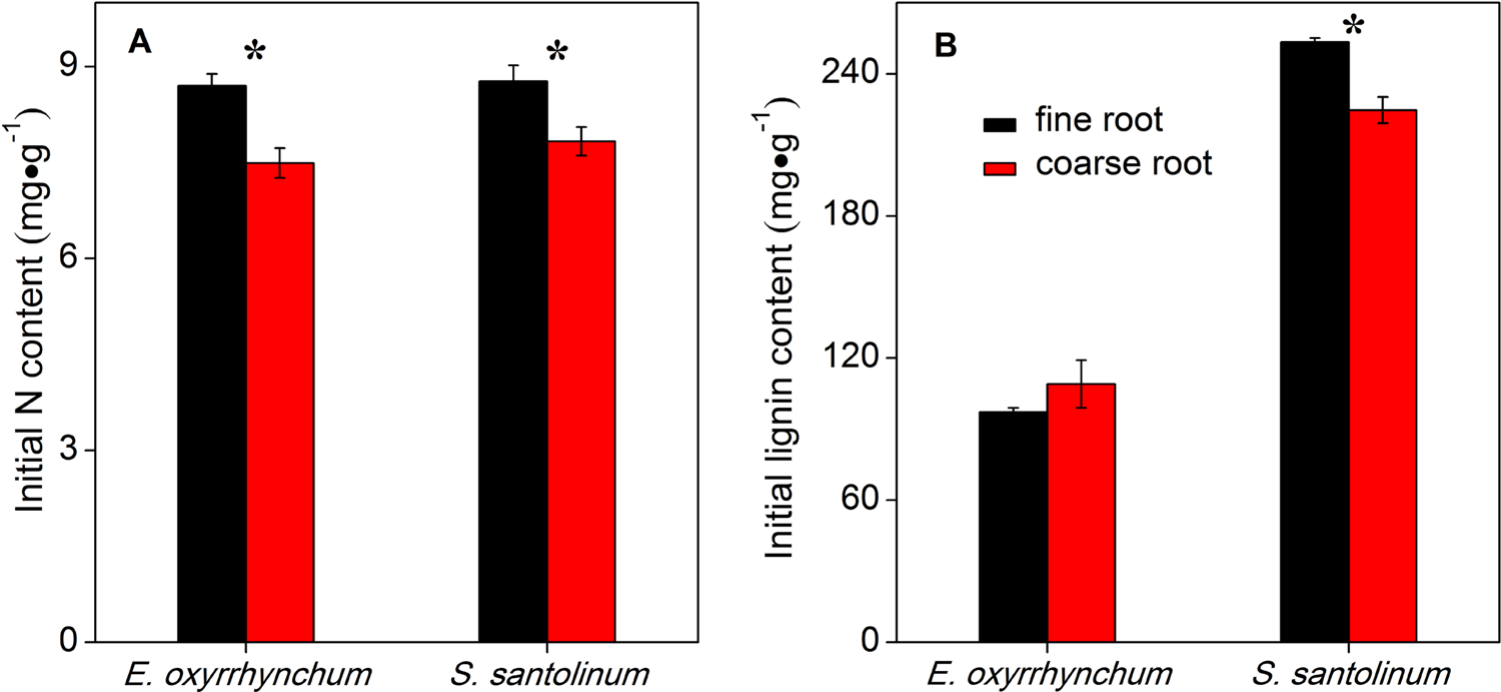
Initial N (A) and lignin (B) concentration of fine and coarse roots from *E*. *oxyrrhynchum* and *S*. *santolinum* in the second experiment (experiment II). * indicates significant differences between fine and coarse roots within each species (*P*<0.05). Vertical bars represent standard errors (n = 3).

Fine and coarse roots differed significantly in their initial N and lignin contents. The initial N content was higher in fine than in coarse roots for *E*. *oxyrrhynchum* and *S*. *santolinum*, whereas *S*. *santolinum* fine roots had higher lignin content than coarse roots (Fig 6).

**Fig 7:**
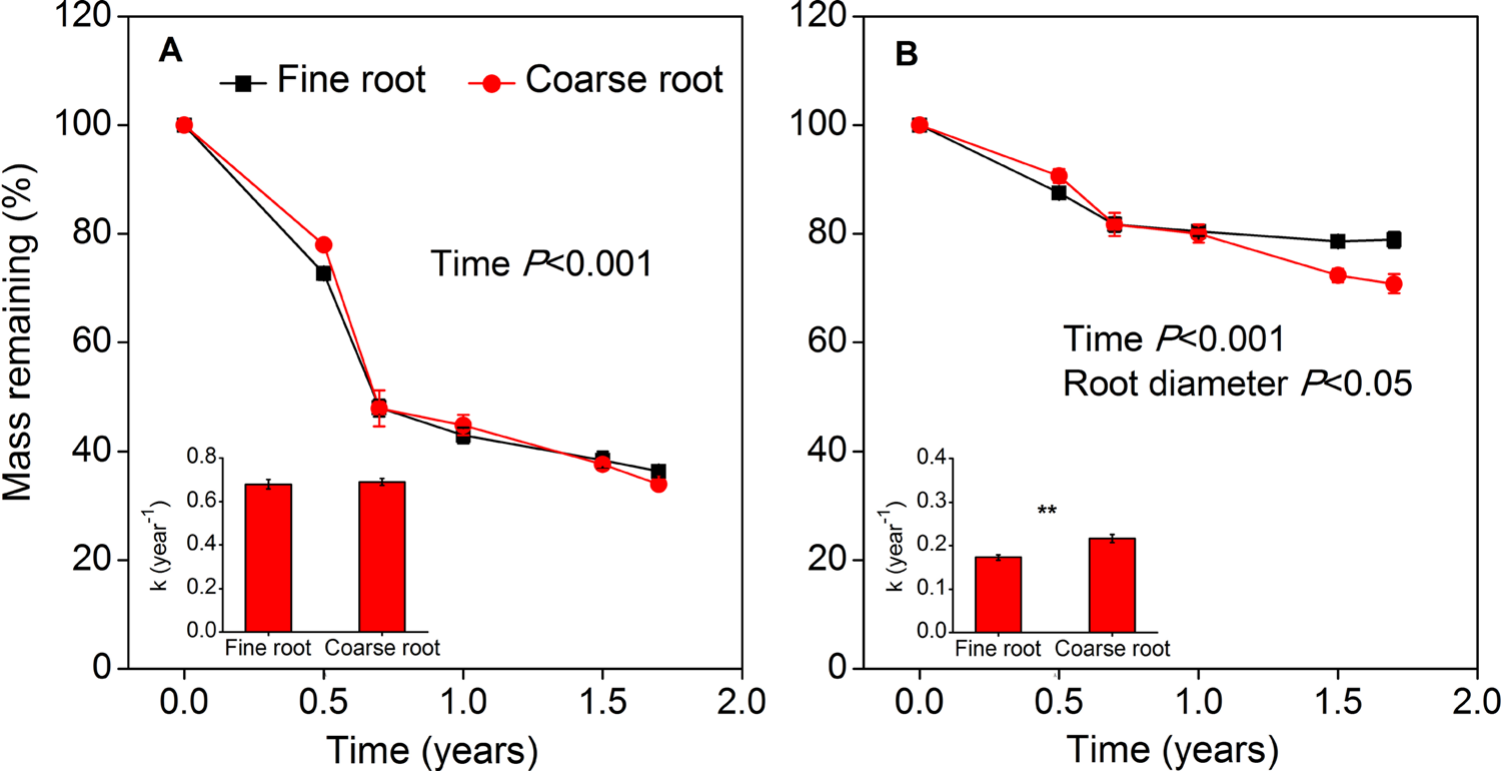
Residual mass for two diameter size classes of *E*. *oxyrrhynchum* (A) and *S*. *santolinum* (B) roots decomposing in the field over a period of 1.7 years (experiment II). Water and N addition treatments have been averaged by root size class within each species. ** indicate significant differences between fine and coarse roots within each species (*P*<0.01). Vertical bars represent standard errors (n = 12). Insets show decomposition rates for fine and coarse roots of *E*. *oxyrrhynchum* and *S*. *santolinum*, respectively.

Residual mass did not differ significantly between diameter classes in *E*. *oxyrrhynchum* (*F* = 0.10, *P* = 0.75), and was significantly lower in fine roots than in coarse roots in *S*. *santolinum* after 1 year of decomposition (*F* = 5.41, *P* = 0.02, Fig 7). Also, fine roots of *S*. *santolinum* had significantly lower decomposition rates than the coarse roots (*F* = 13.69, *P* = 0.001, Fig 7). Consistently, four-way ANOVA showed that the decomposition rate was only significantly affected by species and slightly affected by diameter class (Table 2). There were, however, no interactive effects of species × treatments and root diameter × treatments on rates of decomposition, which indicated that the effects of water and N addition on decomposition dynamics were independent of species and diameter class (Table 2).

**Table 2:** *F* and *P* values from four-way ANOVA for decomposition rate (k), residual N, and P (% of initial mass) during decomposition of root litter (experiment II), with species, root diameter, nitrogen addition, and water addition as main effects.

Source of variation	*k*		N remaining		P remaining	
	*F*	*P*	*F*	*P*	*F*	*P*
Species (S)	1221.74	<0.001	1.95	0.17	71.51	<0.001
Root diameter (D)	3.79	0.06	15.53	<0.001	0.13	0.72
Nitrogen addition (N)	1.71	0.20	0.00	0.99	6.61	0.01
Water addition (W)	0.22	0.64	6.40	0.02	3.89	0.06
S × D	1.35	0.25	0.69	0.41	28.88	<0.001
S × N	0.58	0.45	0.17	0.68	0.06	0.81
D × N	0.19	0.66	0.81	0.37	10.50	<0.01
S × D × N	0.87	0.36	0.24	0.63	0.59	0.45
S × W	1.64	0.21	0.00	0.98	0.52	0.48
D × W	1.15	0.29	9.69	<0.01	2.71	0.11
S × D × W	0.05	0.83	1.84	0.18	0.11	0.74
N × W	4.53	0.04	18.27	<0.001	1.20	0.28
S × N × W	3.10	0.09	3.88	0.06	0.66	0.42
D × N × W	0.06	0.81	0.00	0.95	4.90	0.03
S × D × N × W	1.26	0.27	10.46	<0.01	1.54	0.22

#### Variations in nutrient release between fine and coarse roots

**Fig 8:**
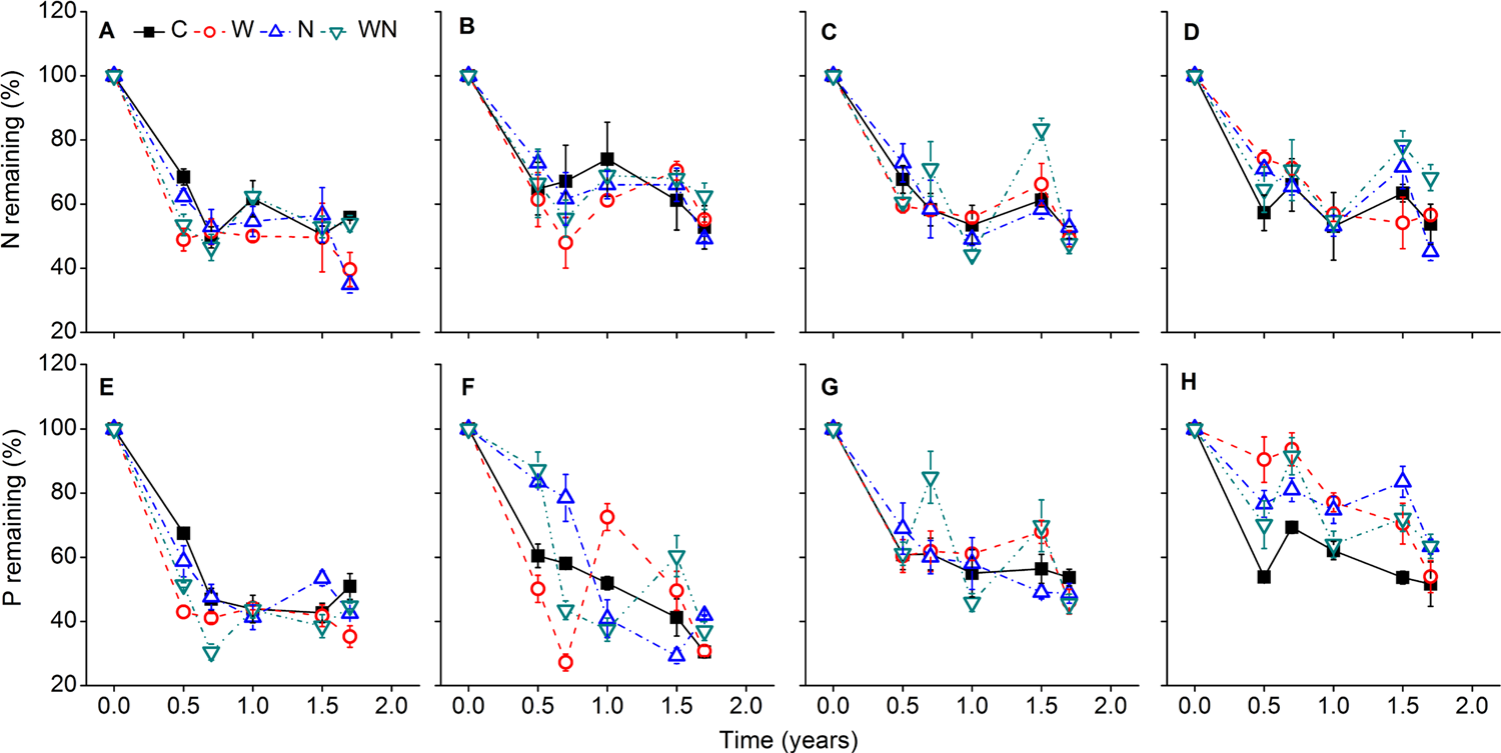
Residual N and P (as percentage of initial content) of fine and coarse roots from *E*. *oxyrrhynchum* and *S*. *santolinum* during the decomposition process under control (C), water (W), nitrogen (N), and water plus nitrogen addition (WN) treatments (experiment II). (A, E) fine and (B, F) coarse roots of *E*. *oxyrrhynchum*; (C, G) fine and (D, H) coarse roots of *S*. *santolinum*. Vertical bars represent standard errors (n *=* 3).

Within the control treatment, residual N and P decreased as decomposition progressed, for both fine and coarse roots, and N addition had no significant effects on loss of N (Fig 8, Table 2). Similar to mass loss, loss of N and P was rapid up to 0.7 years, during which water and N addition had significant effects on P loss of coarse roots. Significantly less litter P remained after 0.7 years of decomposition in the control compared to the N addition treatment for coarse roots. Water and N addition significantly decreased P loss of *S*. *santolinum* coarse roots at 1.7 years compared to control (Fig 8).

### Factors driving herb litter decomposition

**Fig 9:**
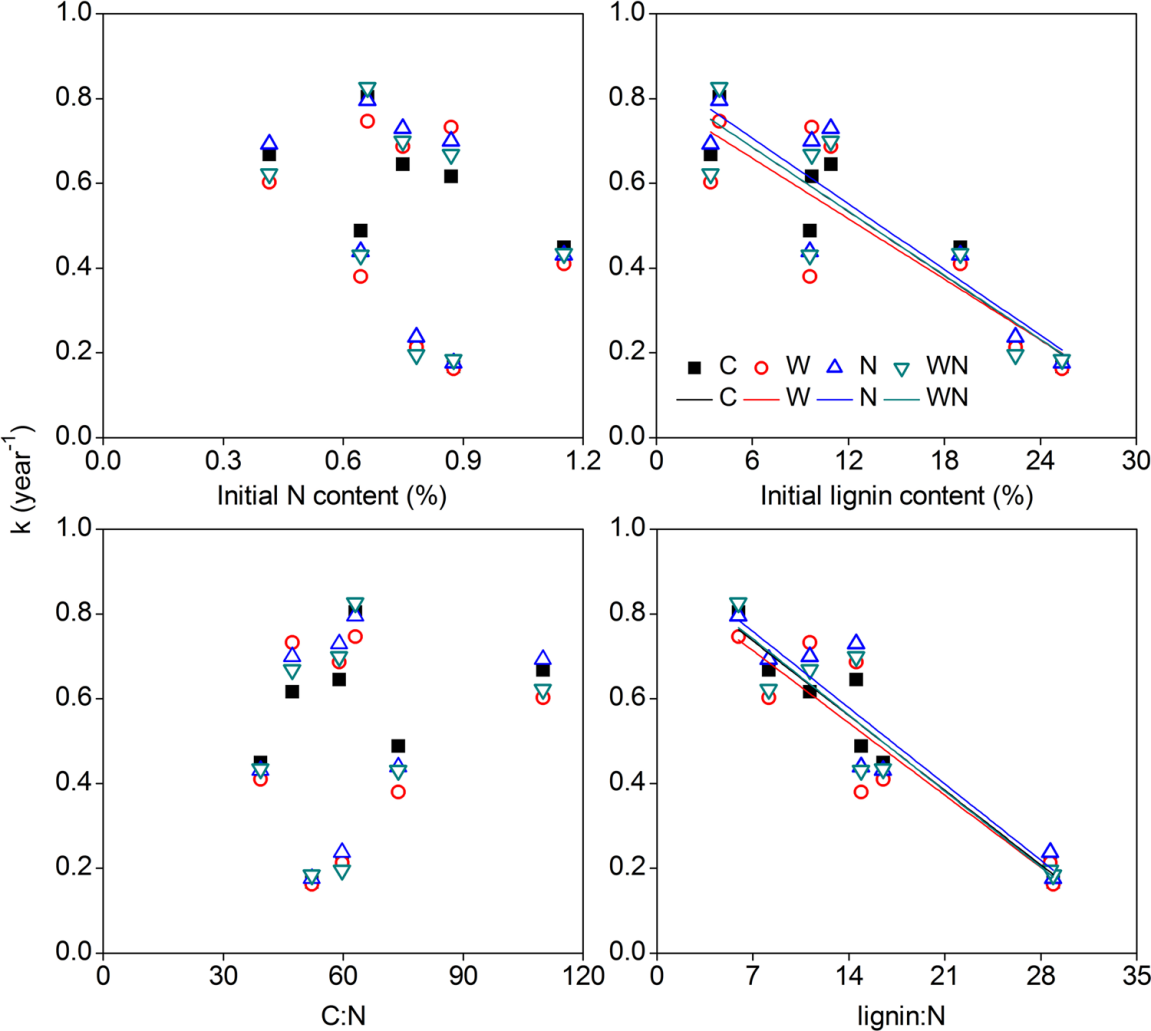
Correlations of decomposition rate (*k*) after 1.7 years with initial N content (A), lignin content (B), C:N ratio (C), and lignin:N ratio (D). Solid line in (B) shows significant linear regression for control (*k* = 0.84–0.03 (% lignin), *R* ^*2*^ = 0.88, *P*<0.01), water addition (*k* = 0.80–0.02 (% lignin), *R* ^*2*^ = 0.72, *P*<0.01), N addition (*k* = 0.86–0.03 (% lignin), *R* ^*2*^ = 0.81, *P*<0.01) and water plus nitrogen treatments (*k* = 0.84–0.03 (% lignin), *R* ^*2*^ = 0.78, *P*<0.01). Solid line in (D) shows significant linear regression for control (*k* = 0.91–0.03 (lignin:N), *R* ^*2*^ = 0.95, *P*<0.001), water addition (*k* = 0.88–0.02 (lignin:N), *R* ^*2*^ = 0.81, *P*<0.01), nitrogen addition (*k* = 0.94–0.03 (lignin:N), *R* ^*2*^ = 0.87, *P*<0.01) and water plus nitrogen treatments (*k* = 0.92–0.03 (lignin:N), *R* ^*2*^ = 0.87, *P*<0.01). Water, nitrogen, and water plus nitrogen treatments did not alter the slope of curves between decomposition rate and initial lignin content (homogeneity of slopes test, *P*>0.05) and lignin:N (homogeneity of slopes test, *P*>0.05).

Decomposition rates of roots after 1.7 years were negatively correlated with initial lignin content (*R* ^2^ *=* 0.88, *P*<0.01) and the lignin:N ratio (*R* ^2^ *=* 0.95, *P*<0.001), but were independent of water and N additions. Other chemical parameters such as the initial N content and C:N ratio, however, were not significantly correlated with decomposition rate (*P*>0.05, Fig 9).

## Discussion

### Effects of water and N addition on root decomposition

Our first hypothesis was not supported by the results, as water and N additions had no significant effects on mass remaining in this experiment (Fig 3). Previous studies have consistently found that neither water nor N addition affect litter decomposition [12,21,38–41]. The lack of response of root litter decomposition to water addition might be related to soil microorganism response to water availability [5]. In mesic deserts (e.g. Chihuanhua), frequent precipitation pulses can maintain considerable soil moisture and accordingly ongoing microbial decomposition [42]. However, in temperate deserts, the soil surface generally experiences a long dry period in summer and, as a consequence, microbial decomposition is typically suspended. The summer water addition treatment of the present study did not lead to subsequent effects on soil water content, as increased soil water content from water addition occurred only in June–August (Fig 1). Although some studies have indicated that water penetration in the soil profile significantly enhances the rate of belowground decomposition [18,43], the majority of precipitation pulses do not penetrate to a depth of 20 cm and affect root decomposition. These findings suggest that water availability to microorganisms is critical for root decomposition [18,42]. Similarly, although N addition increased soil total N content and sporadically impacted nutrient loss during the decomposition process, N enrichment did not affect root mass loss in this study (Figs 2, 4, 5 and 8). These results are consistent with previous studies [21,30,44,45].

We concluded that there are three possible reasons for the lack of response of root decomposition to N addition. First, microbial decomposers might have difficulty accessing external inorganic N, since they tend to use organic N as a N source [44,46]. For instance, N addition stimulated N immobilization in *S*. *subcrassa* (Fig 4) but did not stimulate mass loss during its decomposition process (Fig 3), suggesting that decomposers were accessing inorganic N for some species, even though the stimulation of immobilization was relatively small. However, N addition may not be equivalent to the organic N contained within litter in terms of quantity and quality for microbes. Given the lower energetic costs of using organic N, decomposers may prefer organic N and not respond to inorganic N [44]. Second, there was no significant correlation between decomposition rate and initial N content and C:N ratio (Fig 9), implying N is not a limiting factor for root decomposition. Finally, substrate quality probably regulates the responses of root decomposition to water and N additions [18,41,44,45]. Previous studies have shown that labile substrates can reduce the sensitivity of decomposition to warming and changes in precipitation [41]. The root mass loss of all species (except for *S*. *santolinum*) consistently reached nearly 50% after 0.7 years (Fig 3). Thus, because most labile substrates are utilized directly by decomposers or leached out, effects of external N on decomposition may be obscured [15,19,41,47,].

### Effects of tissue chemistry on root decomposition

The decomposition rate was negatively correlated with initial lignin content and the lignin:N ratio. Roots of *S*. *subcrassa* had low initial lignin content (39.4 mg g^–1^) and the lowest values of lignin:N (i.e. 6), and they showed the fastest decomposition rate among the species (Table 1, Fig 3). Although many chemical characteristics of litter are used to predict decomposition rate, the proportion of the recalcitrants (e.g. lignin) in litter or the lignin:nutrient ratio are generally considered the critical factors in determining root decomposition [11,18,28,48–51]. For instance, the lignin:N ratio can explain the variations of fine root decomposition between C_3_ and C_4_ plants, as well as between forbs and legumes [48]. At the regional scale, initial lignin:N ratio can explain 15% of the variation in root decomposition rates in grasslands and 11% in forests [49]. Also, lignin may be an especially dominant factor in root decomposition in tropical forests and desert watersheds [11,50].

These results emphasized the important role of tissue chemistry in driving litter decomposition [51–54]. We observed a decline in decomposition rates with increasing lignin content and a higher lignin:N ratio of root litter. The detected patterns may reflect that the lignin content and the lignin:N ratio appear to be the best predictors of root decomposition.

Although the C:N ratio has been reported as the critical determinant in decomposition globally [12], no negative correlation between C:N ratio and decomposition rates was found in our study, possibly due to the small variation of initial C:N ratio in these species. The initial C:N ratio was within the range of 39–110 for the six herbs in this study, and identifying a common pattern of functional traits and decomposition would require more samples originating on larger scales. Therefore, regardless of the low initial C:N ratio of desert species, root decomposition was mainly influenced by recalcitrant compounds (e.g. lignin) rather than by the C:N ratio as in temperate deserts.

Aside from the chemical composition of roots, the root diameter is another key factor that governs the decomposition and nutrient dynamics of roots, because it integrates both chemical and physical properties associated with root development [12,21,55]. Fine roots of *S*. *santolinum*, with high N content, decomposed slower than coarse roots, and decomposition rates did not differ significantly between root diameters in *E*. *oxyrrhynchum* (Figs 6 and 7). The results did not support the second hypothesis that fine roots decompose faster than coarse roots, as has been observed by other studies [20,56,57]. Previous studies have shown that initial litter C quality and N content are major factors influencing differences in decomposition rates between root diameters [11,12,19]. Fine roots generally have lower C quality (e.g. high lignin content) but higher N concentration than coarse roots [21]. However, our data suggest that lignin content did not differ significantly between root diameters in *E*. *oxyrrhynchum* (Fig 6). Although the fine roots of *E*. *oxyrrhynchum* had a higher N content than the coarse roots, the higher N content did not result in significantly different decomposition rates. In contrast, the decomposition rates were faster for coarse roots in comparison to fine roots in *S*. *santolinum*, and thereby the relationship between root diameters and decomposition could be explained by the inverse correlation between lignin content and root diameter. Thus, we suspect that C compounds (such as lignin), rather than N concentration, were the major drivers of the slow decomposition rate in fine roots in our study.

Also, other factors such as morphological characteristics or the structure of litter (e.g. architecture and anatomy) may be responsible for the differences in rates of root decomposition among species and between diameter classes [19,20]. Although we did not directly assess the above-mentioned factors in different root materials, previous studies have indicated that morphology is uniquely important for root decomposition [19]. However, given that the physical and chemical properties of roots may interact in influencing root decomposition, further investigations are required to evaluate the integrated importance of the effects of physical and chemical properties on root decomposition.

## Conclusions

Our study showed that root decomposition was dependent on the initial lignin content and the lignin:N ratio across species and diameter classes. Small and temporary water addition treatments as well as N addition treatments did not have significant effects on the decomposition rates of root litter, indicating that microbial activity could not possibly be stimulated by temporary increases in soil water and N. This study helps explain some potential mechanisms by which the root decomposition of desert herbs responds to climate change.
